# Ophiopogonin D mitigates doxorubicin-induced cardiomyocyte ferroptosis through the β-catenin/GPX4 pathway

**DOI:** 10.3389/fphar.2025.1586937

**Published:** 2025-05-23

**Authors:** Yanping Lei, Lewu Xu, Rui Liu, Yue Zhao

**Affiliations:** ^1^ Institute of Cardiovascular Disease, Key Laboratory for Arteriosclerology of Hunan Province, Hunan International Scientific and Technological Cooperation Base of Arteriosclerotic Disease, Hengyang Medical College, University of South China, Hengyang, Hunan, China; ^2^ The First Affiliated Hospital, Department of Cardiology, Hengyang Medical School, University of South China, Hengyang, Hunan, China

**Keywords:** ophiopogonin D, doxorubicin, β-catenin, GPX4, ferroptosis

## Abstract

**Background:**

The chemotherapeutic agent doxorubicin has the side effect of inducing injury to cardiomyocytes. Ferroptosis plays an essential role in the onset and progression of cardiac injury. Ophiopogonin D is considered the active component of the Chinese herbal medicine Mai Dong, which is commonly used for the treatment of cardiovascular diseases. This study investigates the impact of ophiopogonin D on doxorubicin-induced cardiomyocyte ferroptosis by focusing on the β-catenin/GPX4 signaling pathway.

**Methods:**

Mice were injected intraperitoneally with doxorubicin (10 mg/kg) to create a model of cardiotoxicity. Cardiomyocytes exposed to doxorubicin (1 μM) were treated with ophiopogonin D (5 μM). Western blotting was used to detect β-catenin, FTH1, and GPX4. Malondialdehyde (MDA), glutathione (GSH), and Fe^2+^ levels were measured using biochemical assays. In addition, GPX4 expression was detected by immunohistochemistry and immunofluorescence staining. Mitochondrial injury was examined by transmission electron microscopy. Chromatin immunoprecipitation (ChIP) combined with dual-luciferase reporter gene assay was used to analyze the interaction between β-catenin and the promoter of the *GPX4* gene.

**Results:**

Doxorubicin inhibited β-catenin activity and GPX4 expression, promoting cardiomyocyte ferroptosis *in vitro* and *in vivo*. Ophiopogonin D increased β-catenin expression and promoted GPX4 expression, thereby inhibiting doxorubicin-induced ferroptosis in cardiomyocytes. Moreover, β-catenin overexpression enhanced GPX4 expression and alleviated homocysteine-induced ferroptosis in cardiomyocytes. Furthermore, results from the ChIP and dual-luciferase reporter assays indicated that GPX4 acted as a target gene of β-catenin.

**Conclusion:**

Ophiopogonin D inhibits cardiomyocyte ferroptosis induced by doxorubicin by restoring the β-catenin/GPX4 signaling pathway.

## 1 Introduction

Despite the fact that chemotherapy agents have notably enhanced the survival rate of cancer patients, patients concurrently suffer from the cardiotoxicity associated with chemotherapy (Miller et al., 2019). Cardiotoxicity is a common adverse effect of chemotherapy agents and poses potentially fatal risks to cancer patients (Singh et al., 2023). Anthracycline antineoplastic drugs are currently among the most commonly used chemotherapy drugs. Doxorubicin is a representative chemotherapeutic agent that is widely recognized for its efficacy; however, doxorubicin’s cardiotoxicity correlates strongly with cumulative dosing, resulting in irreversible cardiac damage (Renu et al., 2018). The currently available cardioprotective drugs encompass angiotensin-converting enzyme inhibitors/angiotensin receptor blockers (ACEI/ARB), beta-blockers, and statins. These medications demonstrate some therapeutic efficacy in preventing cardiac injury induced by doxorubicin; however, the efficacy of these agents remains limited (Dadson et al., 2022; Keshavarzian et al., 2023). At present, it is imperative to elucidate specific pathological mechanisms underlying doxorubicin-elicited cardiac impairment and develop more effective protective therapies.

Ferroptosis is a distinct form of regulated cell death characterized by the generation of lipid peroxides and the accumulation of intracellular Fe^2+^ (Dixon et al., 2012). Ferroptosis serves as the main mechanism underlying doxorubicin-caused myocardial injury, which is manifested as the accumulation of Fe^2+^ and the inhibition of glutathione peroxidase 4 (GPX4) expression (Tadokoro et al., 2020). As a key negative regulator of ferroptosis, GPX4 is a crucial enzyme that inhibits lipid peroxidation and reduces lipid reactive oxygen species (ROS) through the utilization of glutathione (GSH) (Zhou et al., 2024). The suppression of intracellular iron overload exerts a protective effect against doxorubicin-induced myocardial injury (Fang and Wang, 2019).

The highly conserved β-catenin signaling has garnered attention for its role in response to stress, including injury and inflammation. When Wnt ligands bind membrane receptors, they prevent β-catenin degradation, resulting in its accumulation in the cytoplasm and the subsequent activation of target gene transcription (Reya and Clevers, 2005). As a highly conserved signaling molecule, activated β-catenin signaling promotes cell proliferation and facilitates repair in response to stresses such as inflammation and injury (Masuda and Ishitani, 2017; Valenta et al., 2012; Zuo et al., 2023). β-catenin signaling contributes to the development of the heart (Gessert and Kühl, 2010). In the heart, β-catenin signaling is generally sustained at a low basal level, which is still vital for sustaining heart function.

Shenmai injection, an herbal extract, has demonstrated cardioprotective effects in clinical practice (Lin et al., 2021). It exhibits potential efficacy in mitigating doxorubicin-induced cardiotoxicity (Wu et al., 2021). Ophiopogonin D, an active component of the Shenmai injection, has demonstrated efficacy in protecting the cardiovascular system (Chen et al., 2024).

Based on the emerging evidence, we aim to explore how ophiopogonin D influences doxorubicin-induced ferroptosis through the β-catenin/GPX4 pathway in cardiomyocytes.

## 2 Materials and methods

### 2.1 Animal models

SPF (specific pathogen free) male C57BL/6J mice, aged 8 weeks and weighing 15–20 g, were acquired from Hunan Silaike Jingda Laboratory Animal Co. Ltd. (Changsha, China). Laboratory mice were randomly assigned into six groups (n = 6): 1) the control group; 2) a mouse model of cardiotoxicity (mice were administered doxorubicin at a dosage of 10 mg/kg via intraperitoneal injection on the sixth day); 3) a mouse model of cardiotoxicity treated with daily ophiopogonin D from the first day (10 mg/kg by gavage); 4) mice treated with ophiopogonin D alone; 5) a mouse model of cardiotoxicity infected with AAV-β-catenin (5*10^11^ adeno-associated virus-β-catenin were suspended in 100 μL saline and administered via the tail vein 2 weeks prior to the doxorubicin injection); and 6) a mouse model of cardiotoxicity infected with AAV-control. On the 11th day, mice were euthanized, and heart tissues were extracted for a series of tests. All animal experiments had been approved by the Animal Ethics Committee of the University of South China, located in Hengyang, China.

### 2.2 Cell culture

Neonatal mouse primary cardiomyocytes were isolated following the method described previously (Zhao et al., 2021). The proportion of primary cardiomyocytes in the isolated cell pool was over 95%, as evidenced by immunostaining for α-actin. Cardiomyocytes were cultivated in DMEM/F12 medium with 10% bovine serum. Primary cardiomyocytes underwent serum starvation for 12 h prior to a variety of treatments. Cardiomyocytes were treated with doxorubicin (1 μM) for 46 h. Ophiopogonin D (5 μM) was administered as a treatment, and the incubation time was 48 h. Then, cells were harvested for immunofluorescence staining, electron microscopy examination, and Western blotting.

### 2.3 Cell transfection with plasmids or siRNAs

Cardiomyocytes were cultivated on 6-well plates. Cells were prepared for transfection after the cell confluence reached approximately 80%. Cardiomyocytes were transfected using Lipofectamine with pcDNA3.1-β-catenin (2.5 μg) or pcDNA3.1-GPX4 (2.5 μg). A total of 75 pmol of small interfering RNA targeting β-catenin (β-catenin-siRNA) was incubated with liposomes for 20 min at room temperature to generate a liposome complex. The incubation of cells and the liposome complex lasted for 6 h. Opti-MEM (Gibco, United States) and Lipofectamine 3000 (Invitrogen, United States) were used for the transfection of plasmids and siRNAs. The siRNA sequences were as follows: the β-catenin–siRNA sequence was 5′-GCC​UCU​GAU​AAA​GGC​AAC​UTT-3′, and the β-catenin scramble sequence was 5′-CAG​UAC​UUU​UGU​GUA​GUA​CAA-3’.

### 2.4 Gene delivery mediated by adeno-associated virus vectors

The adeno-associated virus (AAV)-CMV-β-catenin was purchased from Cyagen Biosciences Inc. The plasmids pHBAAV-CMV-β-catenin-3flag, pAAV-RC, and pHelper were co-transfected into HEK293T cells to package the AAV-β-catenin vector. A total of 5*10^11^ AAV particles were diluted in 100 μL saline and intravenously injected into mice. Mice were infected with AAV infection 2 weeks prior to the injection of doxorubicin.

### 2.5 Western blotting

Western blotting for protein quantification was conducted following a standard procedure. Heart tissue and cells were homogenized using the lysis solution from Beyotime Biotechnology. The primary antibodies were as follows: anti-β-catenin (610154; BD Biosciences), anti-active β-catenin (#19807, Cell Signaling Technology), anti-GPX4 (Ab125066, Abcam), anti-FTH1 (Ab183781, Abcam), and anti-β-actin (AA128, Beyotime Biotechnology). The levels of protein expression were normalized using β-actin.

### 2.6 Histology and immunohistochemical analysis

The paraffin sections were prepared according to the protocol, as previously described. Heart sections were immunostained for target proteins using primary antibodies against β-catenin (610154, BD Biosciences, CA) and GPX4 (Ab125066, Abcam). All images were acquired on a bright-field microscope (Nikon).

### 2.7 Immunofluorescent staining

The primary cardiomyocytes from neonatal mice were seeded on the coverslips. Cardiomyocytes were fixed in 4% methanal buffer for 20 min, then permeabilized for 15 min using 1% Triton X-100, followed by 30-min blocking with 10% bovine serum. The slides were incubated overnight with the specified primary antibodies, followed by incubation with Cy-3-conjugated secondary antibodies (A0516, Beyotime Biotechnology). The nuclei were then stained with DAPI for visualization. All micrographs were captured using a Leica fluorescence microscope.

### 2.8 TEM detection

Transmission electron microscopy (TEM) was employed to observe mitochondrial injury in cells and tissues. Small cubic pieces of myocardium (1–2 mm^3^) and cardiomyocytes were sequentially fixed with glutaraldehyde (2.5%) and osmic acid (1%). Subsequently, the samples were embedded, sectioned, and double-stained with uranium acetate (3%) and lead citrate. Ultimately, the mitochondria were examined using a transmission electron microscope (Hitachi).

### 2.9 Echocardiography

The VisualSonics Vevo 2100 Imaging System (VisualSonics Vevo2100 Imaging system, Toronto, Ontario, Canada), equipped with an MS400 probe, was used for echocardiography. Mice were lightly anesthetized with oxygen and 3% isoflurane at a flow rate of 1 L/min. Short-axis views of the left ventricle were captured at the level of the mid-papillary, and a two-dimensional image was recorded for three sequential cardiac cycles.

### 2.10 GSH assay

The intracellular levels of GSH were measured using a kit (#A006-2, Jiancheng), according to the manufacturer’s protocol. The level of GSH was tested using a spectrophotometer at 420 nm. The GSH levels were normalized to the total protein concentration.

### 2.11 Labile iron assay

Labile iron concentrations in cardiomyocytes and cardiac tissue were assessed using a kit (#ab83366, Abcam), following the instructions of the manufacturer.

### 2.12 Lipid ROS assay

The level of lipid ROS was tested using BODIPY581/591 C11, a fluorescent probe (D3861, Invitrogen, United States). Cardiomyocytes were transfected with pcDNA3.1–β-catenin, then stimulated with doxorubicin, and incubated with BODIPY581/591 C11 at a concentration of 10 μM for 30 min in the dark. Cells were visualized using the fluorescence microscope (Leica, Wetzlar, Germany). Malondialdehyde (MDA) was quantified via thiobarbituric acid, and the MDA assay kit was purchased from Beyotime (Nanjing, China).

### 2.13 Chromatin immunoprecipitation

The pcDNA3.1–β-catenin plasmid was used to transfect H9c2 cells. After 48 h, cells were fixed in 4% formaldehyde buffer to facilitate protein–DNA crosslinking. ChIP was carried out using the SimpleChIP Plus Kit (Cat. 9005, Cell Signaling). The antibodies against β-catenin (Ab32572, Abcam), RNAP Ⅱ (RNA polymerase Ⅱ), and rabbit IgG were added for incubation overnight at 4°C. Subsequently, protein A-agarose was introduced for incubation for 1 h. Then, the extracted DNA was used as the template for PCR amplification. The primer pair designed for the *GPX4* gene promoter included the forward primer 5′-AAG​CCA​GGT​TTC​CTT​GTG​TG-3′ and the reverse primer 5′-ATG​CCT​GTG​ACT​GTA​CAT​GC-3’.

### 2.14 RT-PCR

Total RNA in cells was extracted using the TRIzol reagent. An amount of 2 μg of RNA was added as a template to a 20 µL reverse transcription reaction system (RK20433, ABclonal) to synthesize the first-strand cDNA. RT-PCR was conducted using an Applied Biosystems 2720 Thermal Cycler (United States). The 25 µL reaction system consisted of 12.5 µL Taq PCR Mix (RK20608, ABclonal), 1 µL RT product, and 0.2 μM sense and antisense primers. In brief, after 2 min of initial incubation at 95°C, the PCR amplification contained 35 cycles, including 30 s of denaturation at 95°C, 30 s of annealing at 60°C, and 60 s of extension at 72°C. The levels of mRNA were reported with normalization to β-actin. The primer pair designed for GPX4 PCR was listed as follows: sense primer 5′-CAA​CCA​GTT​CGG​GAG​GCA​GGA​G-3′ and antisense primer 5′-TGG​GCT​GGA​CTT​TCA​TCC​ATT​TC-3’.

### 2.15 Dual-luciferase reporter gene assay

The dual-luciferase reporter gene assay kit and reporter gene plasmids (pGL6-TA and pRL-SV40-C) were purchased from Beyotime Biotechnology. Both the pGL6-TA-GPX4 promoter and pRL-SV40-C were co-transfected into HEK293T cells along with pcDNA3.1–β-catenin. After 48 h of transfection, the cells were subjected to lysis and centrifugation to obtain the supernatant. Samples were loaded onto a 96-well microplate, and fluorescence intensity was determined using a luminometer (BioTek, Synergy, United States). Luciferase activity was evaluated by measuring the intensity of firefly fluorescence, which was standardized to the intensity of Renilla fluorescence.

### 2.16 [Ca^2+^]i measurement

The intracellular Ca^2+^ concentration ([Ca^2+^]i) was assessed using the Ca^2+^ indicator (Fluo-3/AM, DOJINDO, Japan), according to the manufacturer’s instructions. In detail, after indicated treatments, cardiomyocytes were incubated with fluo-3/AM (2.5 μM) at 37°C for 30 min in the dark and then rinsed with Ca^2+^-free and Mg^2+^-free D-Hank’s solution to remove remaining fluo-3/AM. Following scanning for approximately 2 min to record the baseline of intracellular Ca^2+^, the cells were exposed to calcium chloride (1.8 mmol/L), and the dynamics of intracellular Ca^2+^ were recorded for 8 min. A series of images were captured using a laser scanning confocal microscope (LSCM, Nikon A1, Japan) at an excitation wavelength of 488 nm with 5 s intervals. Image analysis was performed using Nikon Confocal software by randomly selecting six cells to analyze their dynamic fluorescence intensity. Data were presented as baseline Ca^2+^ combined with the amount of Ca^2+^ released.

### 2.17 Mitochondrial membrane potential assay

The mitochondrial membrane potential was measured using a JC-1 kit (C2006) purchased from Beyotime Biotechnology. In brief, 1 mL of staining working solution was introduced and thoroughly mixed. Then, cells were put in a cell incubator for 20 min. Following incubation, JC-1 staining buffer was used to wash the cells twice. Thereafter, the fluorescence images in green and red were captured under excitation wavelengths of 525 nm and 490 nm.

### 2.18 Statistical analyses

All data were expressed as the mean ± SEM in our study. Statistical analysis was performed using GraphPad Prism (La Jolla, CA). The comparison among groups was implemented using a one-way ANOVA. Statistical analyses between two groups were carried out using the Student–Newman–Keuls test. A p-value of <0.05 was considered statistically significant.

## 3 Results

### 3.1 Doxorubicin induced ferroptosis and downregulated the levels of β-catenin and GPX4 in cardiomyocytes

**FIGURE 1:**
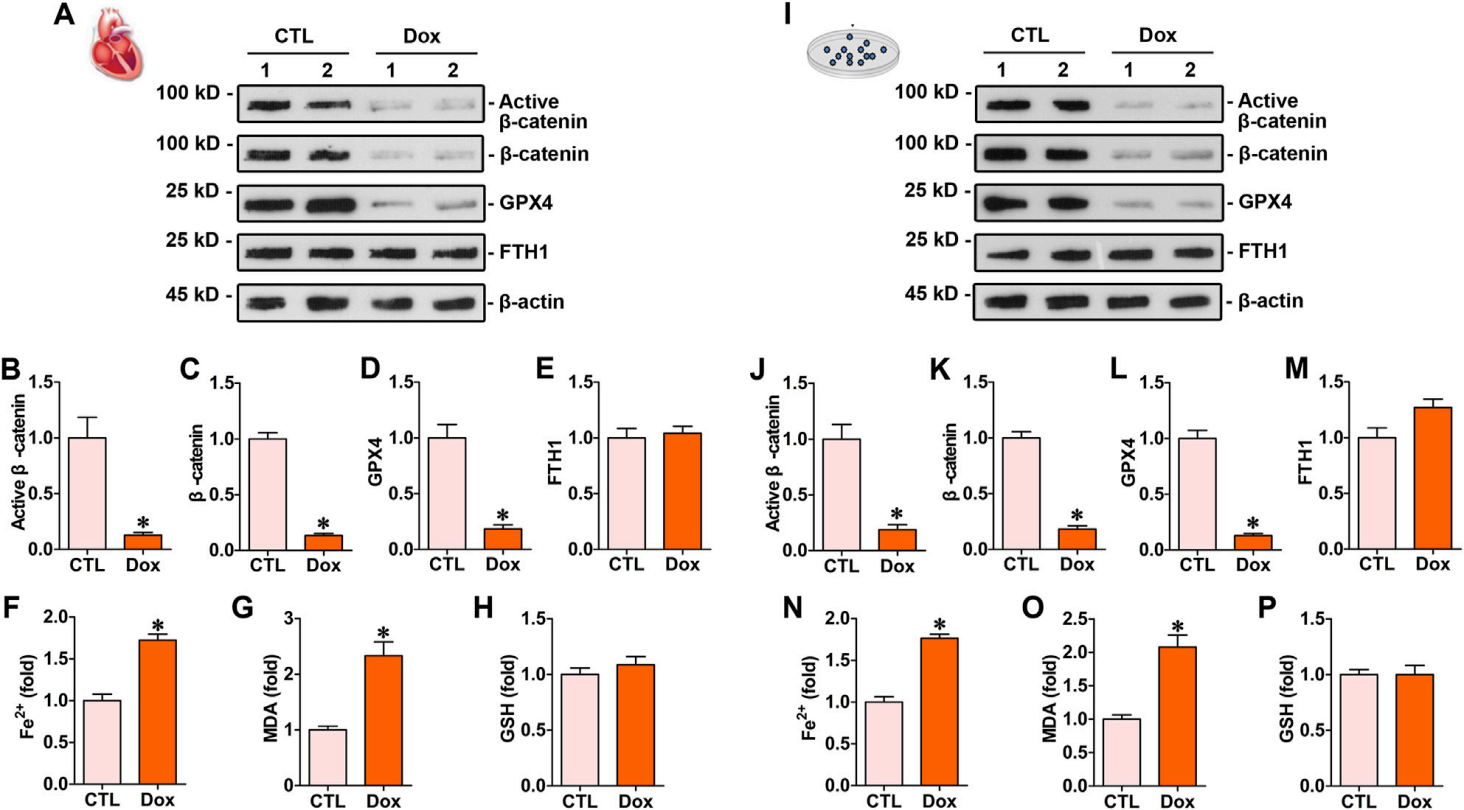
Doxorubicin downregulated β-catenin signaling and GPX4 expression levels while increasing Fe^2+^ and MDA levels in the myocardium both *in vitro* and *in vivo*. **(A)** Western blot analysis showed the expression levels of protein, including active β-catenin, total β-catenin, GPX4, and FTH1, in the hearts of mice administered with doxorubicin. **(B–E)** Quantitative analysis of active β-catenin, total β-catenin, GPX4, and FTH1 proteins in the indicated groups. Protein levels were expressed as fold induction to the controls. *P < 0.05 vs. the controls. **(F–H)** Quantitative determination of Fe^2+^, MDA, and GSH on the colorimetric microplate reader. *P < 0.05 vs. the controls. **(I)** Western blot showed the levels of active β-catenin, total β-catenin, GPX4, and FTH1 in cardiomyocytes subjected to doxorubicin. **(J–M)** Quantitative analysis of active β-catenin, total β-catenin, GPX4, and FTH1 protein levels in the indicated groups. Protein levels were expressed as fold induction to the controls. *P < 0.05 vs. the controls (n = 6). **(N–P)** Quantitative determination of Fe^2+^, MDA, and GSH in cardiomyocytes. *P < 0.05 vs. the controls (n = 6).

The animal model of doxorubicin-induced cardiotoxicity was established by injecting doxorubicin (10 mg/kg, i.p). The myocardial protein levels of β-catenin and GPX4 in mice subjected to doxorubicin stimulation were markedly lower than those in the control group, whereas the expression of FTH1 remained unchanged (Figures 1A–E). Biochemical assay results indicated that myocardial Fe^2+^ and MDA levels in mice subjected to doxorubicin injection were considerably higher than those in the control mice (Figures 1F,G). *In vitro* experiments showed that doxorubicin induced the downregulation of β-catenin and GPX4 (Figures 1I–L), simultaneously increasing Fe^2+^ and MDA in cardiomyocytes compared to the controls (Figures 1N,O). Both *in vivo* and *in vitro* experiments demonstrated that doxorubicin had no impact on the levels of FTH1 and GSH in cardiac tissues (Figures 1E,H,M,P).

### 3.2 Ophiopogonin D inhibited ferroptosis and restored β-catenin and GPX4 expression in the hearts of doxorubicin-treated mice

**FIGURE 2:**
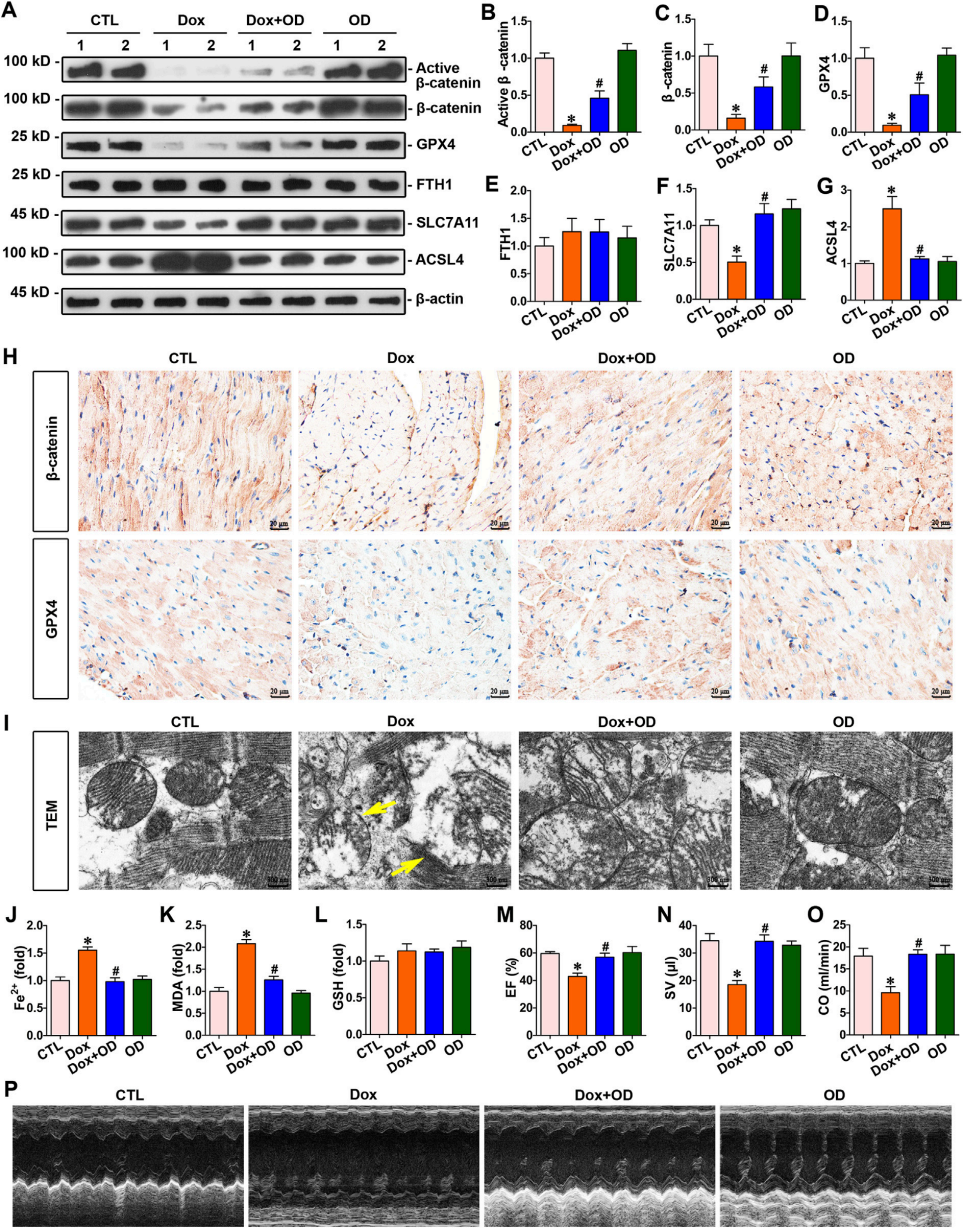
Ophiopogonin D restored β-catenin and GPX4 expression levels, inhibited ferroptosis, and improved cardiac function in the hearts of mice exposed to doxorubicin. **(A)** Western blot analysis revealed the expression levels of active β-catenin, total β-catenin, GPX4, FTH1, SLC7A11, and ACSL4 in the hearts of each group. **(B–G)** Quantitative analysis of protein levels in Figure 2A are displayed. **(H)** Representative micrographs showed β-catenin and GPX4 staining in the hearts of the specified mice. Upper panel, immunostaining for β-catenin in mouse heart tissue as indicated; bottom panel, immunostaining for GPX4 in the given groups as indicated. Scale bar, 20 μm. **(I)** Representative TEM images showed mitochondrial damage in the hearts of mice in the indicated groups. The yellow arrows indicated injured mitochondria. Scale bar, 300 nm. **(J–L)** Quantitative determination of Fe^2+^, MDA, and GSH in the indicated groups on a colorimetric microplate reader. **(M–O)** Statistical quantification of EF, SV, and CO among the groups. **(P)** Representative echocardiograms along the short axis. *P < 0.05 vs. the controls; ^#^ *p* < 0.05 vs. doxorubicin stimulation alone (n = 6).

To assess its protective efficacy against cardiac injury induced by doxorubicin, ophiopogonin D was administered orally to doxorubicin-treated mice daily. Western blot analysis showed that ophiopogonin D could induce the upregulation of active β-catenin, β-catenin, and GPX4. However, no significant differences were observed in the FTH1 expression levels among the groups (Figures 2A–E). *SLC7A11* and *ACSL4* are two crucial genes closely associated with ferroptosis. *SLC7A11* suppresses the occurrence of ferroptosis via an antioxidant pathway, whereas *ACSL4* facilitates the process of ferroptosis by augmenting the generation of lipid peroxidation (Chen et al., 2025). Doxorubicin decreased the level of *SLC7A11* and concurrently upregulated the expression of *ACSL4*. In contrast, ophiopogonin D was capable of antagonizing the action of doxorubicin, manifested as upregulating the level of *SLC7A11* and downregulating the expression of *ACSL4*. The aforementioned results indicated that doxorubicin promoted ferroptosis, whereas ophiopogonin D inhibited ferroptosis by counteracting the effect of doxorubicin (Figures 2A,F,G). Further immunohistochemical staining showed a significant reduction in the expression levels of both β-catenin and GPX4 in the myocardium of mice injected with doxorubicin compared to those in the control group. Notably, ophiopogonin D treatment reestablished the expression levels of both β-catenin and GPX4 (Figure 2H). A transmission electron microscope was used to detect mitochondrial morphology. As presented in Figure 2I, doxorubicin induced notable mitochondrial damage (indicated by yellow arrows), whereas ophiopogonin D mitigated this injury. Compared with the concentrations in the doxorubicin-stimulated mice, treatment with ophiopogonin D significantly reduced myocardial concentrations of Fe^2+^ and MDA (Figures 2J,K), did not affect the lelvel of GSH (Figure 2L). Furthermore, echocardiograms revealed that doxorubicin markedly impaired cardiac contractility, as manifested by the decreases in stroke volume (SV), ejection fraction (EF), and cardiac output (CO) (Figures 2M–P).

### 3.3 Ophiopogonin D inhibited ferroptosis and restored the expression of β-catenin and GPX4 against doxorubicin in cardiomyocytes

**FIGURE 3:**
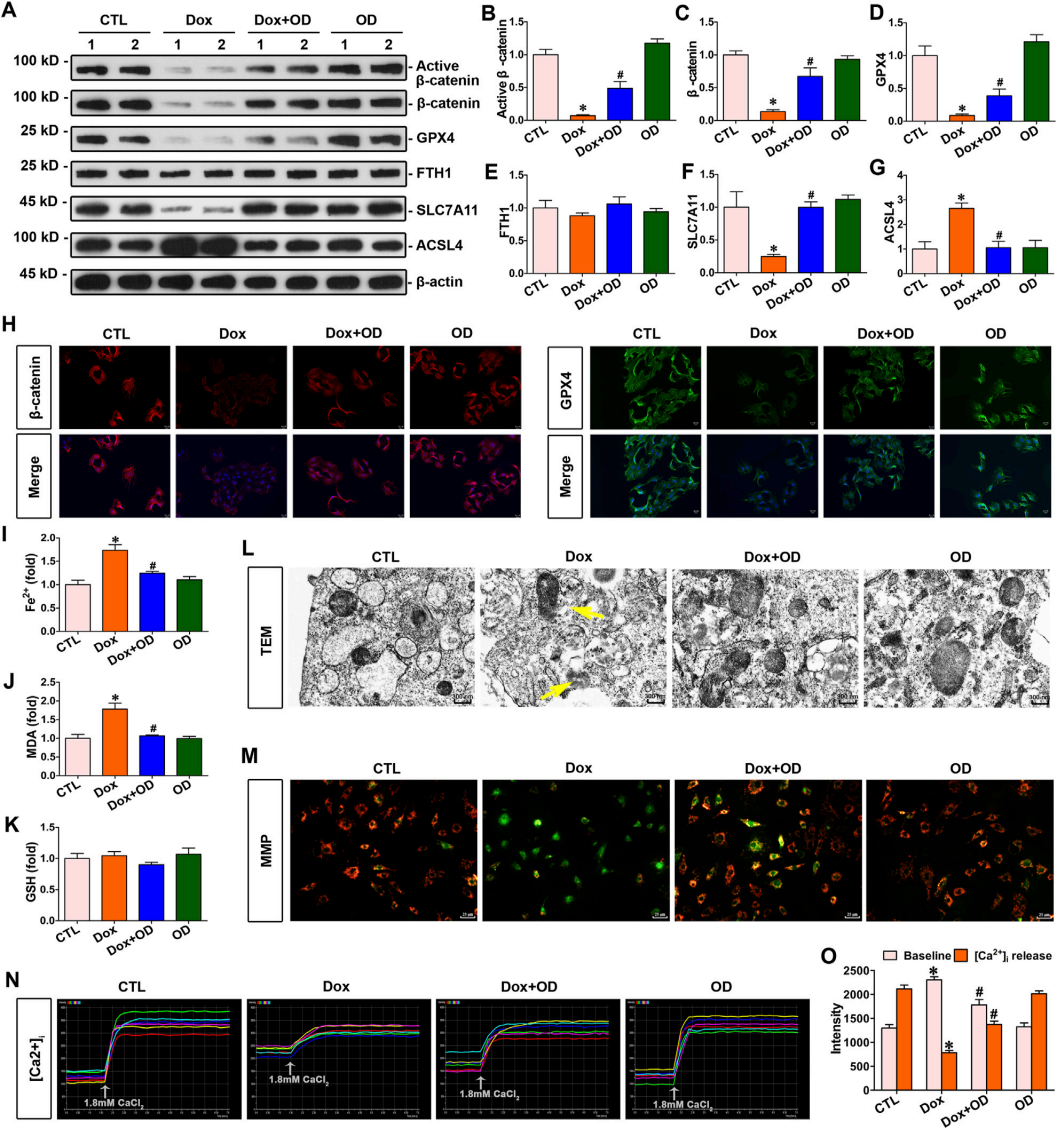
Ophiopogonin D restored β-catenin and GPX4 expression levels, inhibited ferroptosis, and improved mitochondrial function in cardiomyocytes against doxorubicin. **(A)** Western blot analysis revealed the expression levels of active β-catenin, total β-catenin, GPX4, FTH1, SLC7A11, and ACSL4 in the cardiomyocytes of each group as indicated. **(B–G)** Quantitative analysis of the abundance of the specified proteins from Figure 3A is presented for each group. **(H)** Representative micrographs showed staining for β-catenin and GPX4 proteins in cardiomyocytes. The left panel displays immunofluorescent staining of β-catenin in the given groups; the right panel displays immunostaining for GPX4 in cardiomyocytes in the indicated groups. Scale bar, 25 μm. **(I–K)** Biochemical assay of Fe^2+^, MDA, and GSH in the indicated groups on a colorimetric microplate reader. *P < 0.05 vs. the controls; ^#^ *p* < 0.05 vs. doxorubicin stimulation alone (n = 6). **(L)** Representative transmission electron microscope images showed mitochondrial damage in cardiomyocytes of the indicated groups. The yellow arrows indicated injured mitochondria. Scale bar, 300 nm. **(M)** The JC-1 kit was used to assess mitochondrial membrane potential. Red fluorescence indicated normal mitochondrial membrane potential, whereas green fluorescence indicated a decrease in mitochondrial membrane potential. **(N)** Dynamic curve of Ca^2+^ concentration in six cardiomyocytes of the indicated groups. **(O)** Quantitative data for Ca^2+^ levels in each group of Figure 3N. *P < 0.05 vs. the controls; ^#^ *p* < 0.05 vs. doxorubicin stimulation alone (n = 6).

Primary mouse cardiomyocytes were stimulated with doxorubicin (1 μM), and ophiopogonin D (5 μM) was added as a treatment. Western blot analysis indicated that doxorubicin inhibited the expression of active β-catenin, β-catenin, and GPX4 without affecting FTH1 expression. In contrast, treatment with ophiopogonin D restored the abundance of active β-catenin, β-catenin, and GPX4 in cardiomyocytes (Figures 3A–E). Doxorubicin decreased the level of *SLC7A11* and concurrently upregulated the expression of *ACSL4*. In contrast, ophiopogonin D was capable of antagonizing the action of doxorubicin, manifested as upregulating the level of SLC7A11 and downregulating the expression of ACSL4. The aforementioned results indicated that doxorubicin promoted ferroptosis, whereas ophiopogonin D inhibited ferroptosis by counteracting the effect of doxorubicin (Figures 3A,F,G). Doxorubicin significantly induced the upregulation of Fe^2+^ and MDA, while it did not impact the level of GSH in cardiomyocytes. Compared with the doxorubicin-stimulated cardiomyocytes, ophiopogonin D treatment significantly reduced intracellular levels of Fe^2+^ and MDA (Figures 3I–K). Further immunofluorescence staining showed that the expression levels of β-catenin and GPX4 in cardiomyocytes were significantly reduced following doxorubicin stimulation compared to those in the control group. Conversely, treatment with ophiopogonin D counteracted the effects of doxorubicin, increasing β-catenin and GPX4 levels (Figure 3H). Furthermore, transmission electron microscopy images revealed that doxorubicin caused visible mitochondrial injuries and that ophiopogonin D significantly ameliorated these injuries (Figure 3L). Given that mitochondrial membrane potential and intracellular Ca^2+^ flux served as indicators of mitochondrial function, we used the JC-1 kit and Fluo-3/AM fluorescent dye to assess the mitochondrial membrane potential and intracellular calcium flux in cardiomyocytes after the indicated treatments. The augmented green fluorescence in cardiomyocytes stimulated by doxorubicin suggested a decrease in mitochondrial membrane potential, whereas the enhanced red fluorescence in cardiomyocytes treated with ophiopogonin D indicated an elevation of the membrane potential compared to that in the doxorubicin-treated group (Figure 3M). Additionally, doxorubicin conspicuously elevated the baseline Ca^2+^ level within cardiomyocytes while diminishing the Ca^2+^ release within cardiomyocytes in response to calcium chloride stimulation (Figures 3N,O). The abovementioned alterations indicated mitochondrial dysfunction. On the contrary, ophiopogonin D effectively counteracted these alterations induced by doxorubicin.

### 3.4 Increased β-catenin expression attenuated doxorubicin-induced myocardial ferroptosis and increased GPX4 expression

**FIGURE 4:**
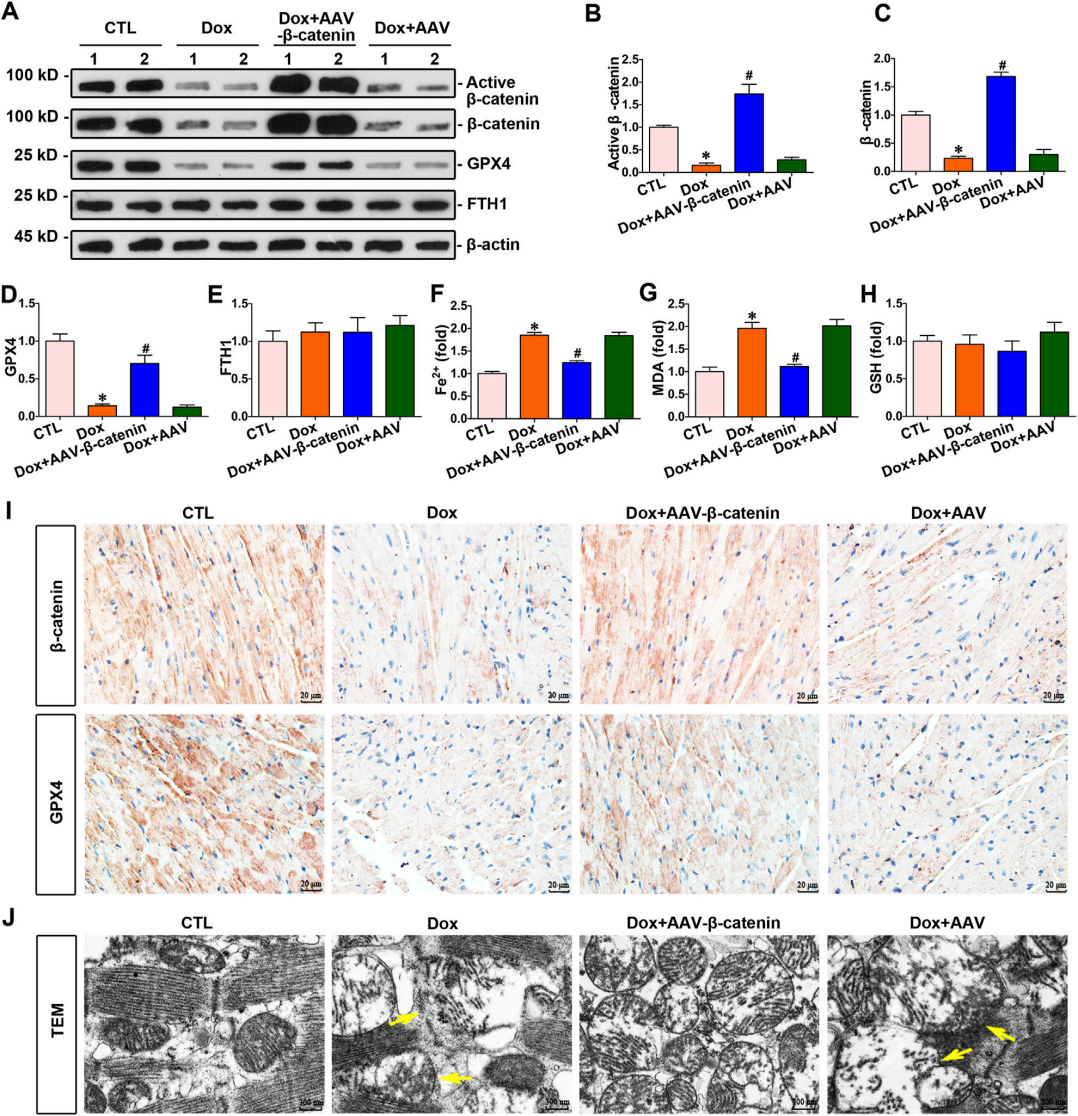
Increased β-catenin expression upregulated the expression of GPX4 and inhibited myocardium ferroptosis against doxorubicin. **(A)** Western blot analysis revealed the expression levels of active β-catenin, total β-catenin, GPX4, and FTH1 in cardiomyocytes of each group. **(B–E)** Quantitative analysis of the abundance of specific proteins in the indicated groups of Figure 4A are displayed. **(F–H)** Quantitative determination of Fe^2+^, MDA, and GSH in the indicated groups on a colorimetric microplate reader. *P < 0.05 vs. the controls; ^#^ *p* < 0.05 vs. doxorubicin stimulation alone (n = 6). **(I)** Representative images showed staining for β-catenin and GPX4 proteins in mouse heart tissue. Upper panel, immunostaining for β-catenin in mouse heart tissue as indicated; bottom panel, immunostaining for GPX4 in the given groups as indicated. Scale bar, 20 μm. **(J)** Representative TEM images showed subcellular structure in the hearts of mice in the indicated groups. The yellow arrows indicated injured mitochondria. Scale bar, 300 nm.

To explore the association between β-catenin and doxorubicin-induced ferroptosis in the myocardium, mice were administered doxorubicin and infected with AAV-β-catenin. Enhanced β-catenin expression increased the expression of GPX4 (Figures 4A–D), simultaneously decreasing the level of Fe^2+^ and MDA in the hearts of doxorubicin-stimulated mice (Figures 4F,G), but did not affected the levels of FTH1 and GSH (Figures 4E,H). Further immunohistochemical staining indicated that the expression levels of both β-catenin and GPX4 were markedly reduced in the myocardium of mice injected with doxorubicin compared to those in the control group, while infection with AAV-β-catenin induced overexpression of β-catenin and restored GPX4 expression suppressed by doxorubicin (Figure 4I). In addition, a transmission electron microscope was used to detect mitochondrial morphology. As presented in Figure 4J, doxorubicin induced notable mitochondrial damage, such as mitochondrial membrane disruption and shrinkage, whereas activated β-catenin signaling mitigated these mitochondrial injuries. In summary, activated β-catenin signaling preserved the heart against doxorubicin-induced ferroptosis, coupled with an elevation of GPX4.

### 3.5 Increased β-catenin expression inhibited doxorubicin-induced cardiomyocyte ferroptosis and upregulated the expression of GPX4

**FIGURE 5:**
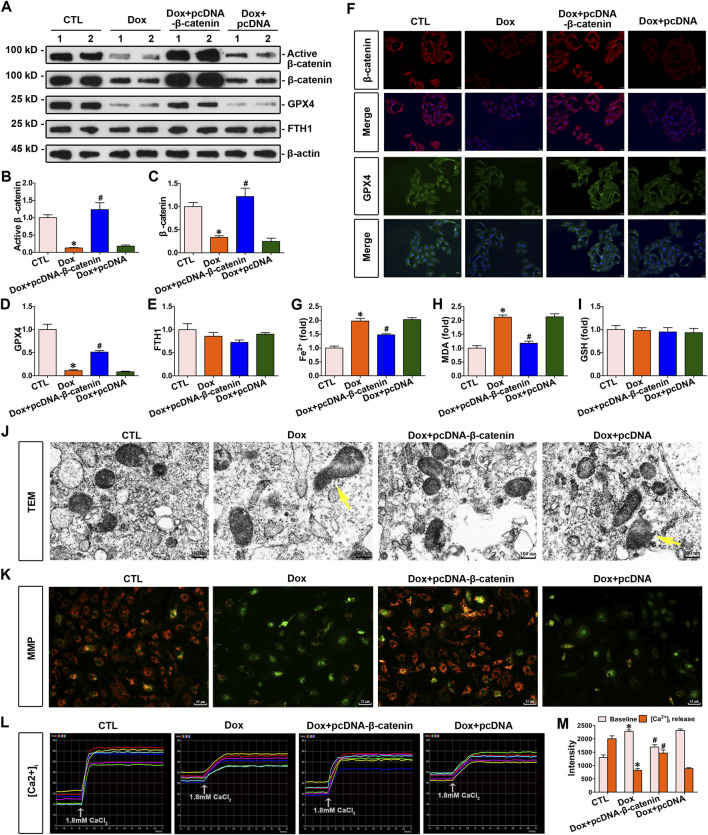
Increased β-catenin expression enhanced the expression of GPX4, inhibited doxorubicin-induced ferroptosis, and improved mitochondrial function in cardiomyocytes. **(A)** Western blot analysis revealed the expression levels of active β-catenin, total β-catenin, GPX4, and FTH1 in the cardiomyocytes of each group. **(B–E)** Quantitative analysis of the abundance of specific proteins in the indicated groups of Figure 5A is displayed. **(F)** Representative micrographs showed staining for β-catenin and GPX4 proteins within myocytes of each group. Scale bar, 25 μm. **(G–I)** Measurement of Fe^2+^, MDA, and GSH in the indicated groups on a colorimetric microplate reader. *P < 0.05 vs. the controls; ^#^ *p* < 0.05 vs. doxorubicin stimulation alone (n = 6). **(J)** Representative transmission electron microscope images showed subcellular structure in cardiomyocytes of the given groups. The yellow arrows indicated injured mitochondria. Scale bar, 300 nm. **(K)** JC-1 kit was used to assess mitochondrial membrane potential. Red fluorescence indicated normal mitochondrial membrane potential, whereas green fluorescence indicated a decrease in mitochondrial membrane potential. **(L)** Dynamic curve of Ca^2+^ concentration in six cardiomyocytes of the indicated groups. **(M)** Quantitative data for Ca^2+^ levels in each group of Figure 5L. *P < 0.05 vs. the controls; ^#^ *p* < 0.05 vs. doxorubicin stimulation alone (n = 6).

To explore the association between β-catenin and doxorubicin-induced ferroptosis in cardiomyocytes, cardiomyocytes were stimulated with doxorubicin and transfected with pcDNA3.1–β-catenin. The elevated expression of β-catenin increased GPX4 expression (Figures 5A–D), simultaneously decreasing the concentrations of Fe^2+^ and MDA in cardiomyocytes (Figures 5G,H). Nevertheless, elevated β-catenin expression did not alter FTH1 or GSH levels in cardiomyocytes (Figures 5E,I). Furthermore, immunofluorescence demonstrated that β-catenin and GPX4 expression levels in cardiomyocytes were significantly decreased following doxorubicin stimulation compared to those in the control group. On the contrary, β-catenin overexpression elevated GPX4 levels in response to doxorubicin (Figure 5F). Furthermore, transmission electron microscopy images revealed that doxorubicin caused visible mitochondrial damage, and elevated β-catenin signaling significantly ameliorated this damage (Figure 5J). Taken together, upregulated β-catenin signaling preserved cardiomyocytes against ferroptosis induced by doxorubicin. Moreover, the cardiomyocyte function assay demonstrated that doxorubicin stimulation conspicuously reduced the mitochondrial membrane potential of cardiomyocytes compared to that in the control group, whereas the overexpression of β-catenin significantly restored the mitochondrial membrane potential of cardiomyocytes under doxorubicin stimulation (Figure 5K). In addition, Ca^2+^ assays indicated a significant increase in baseline Ca^2+^ levels, while Ca^2+^ release in response to calcium chloride stimulation is markedly decreased in cardiomyocytes under doxorubicin stimulation. Conspicuously, elevated β-catenin expression ameliorated Ca^2+^ disorder induced by doxorubicin in cardiomyocytes (Figures 5L,M). Collectively, β-catenin overexpression preserved mitochondrial integrity against doxorubicin.

### 3.6 The upregulation of GPX4 inhibited cardiomyocyte ferroptosis induced by doxorubicin

**FIGURE 6:**
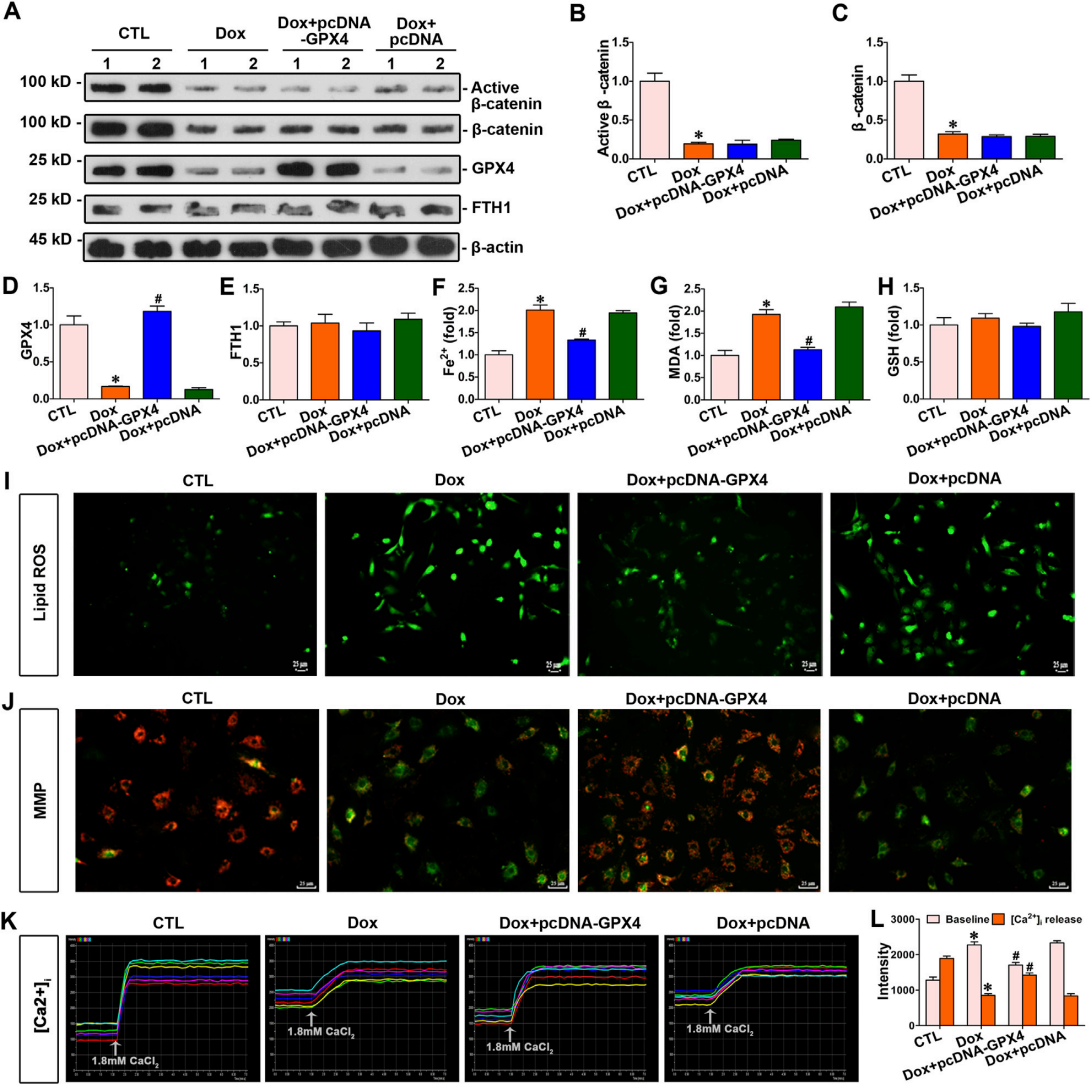
Overexpression of GPX4 inhibited doxorubicin-induced cardiomyocyte ferroptosis and improved mitochondrial function against doxorubicin. **(A)** Western blot analysis revealed the expression levels of active β-catenin, total β-catenin, GPX4, and FTH1 in cardiomyocytes of each group. **(B–E)** Quantitative analysis of the abundance of specific proteins in the indicated groups of Figure 6A is displayed. **(F–H)** Quantitative determination of Fe^2+^, MDA, and GSH in the indicated groups on a colorimetric microplate reader. *P < 0.05 vs. the controls; ^#^ *p* < 0.05 vs. doxorubicin stimulation alone (n = 6). **(I)** Representative images showed staining for lipid ROS with BODIPY581/591 C11. Scale bar, 25 μm. **(J)** Assessment of mitochondrial membrane potential in the indicated groups using the JC-1 kit. **(K)** Dynamic curve of Ca^2+^ concentration in six cardiomyocytes of the indicated groups. **(L)** Quantitative data for Ca^2+^ levels in each group of Figure 6K. *P < 0.05 vs. the controls; ^#^ *p* < 0.05 vs. doxorubicin stimulation alone (n = 6).

To further explore the connection between GPX4 and doxorubicin-induced ferroptosis, myocytes were exposed to doxorubicin and transfected with pcDNA3.1–GPX4 to enhance GPX4 expression. Increased GPX4 expression decreased the concentrations of Fe^2+^ and MDA (Figures 6F,G). Nevertheless, elevated GPX4 expression did not impact the levels of β-catenin, FTH1, and GSH in cardiomyocytes (Figures 6A–E,H). In addition, lipid ROS levels were assessed by the fluorescent probes C11-BODIPY. As presented in Figure 6I, doxorubicin notably elevated lipid ROS levels, but GPX4 overexpression reduced the level of lipid ROS induced by doxorubicin. Thus, upregulated GPX4 preserved cardiomyocytes against doxorubicin-induced ferroptosis. In addition, the cardiomyocyte function assay demonstrated that GPX4 overexpression effectively mitigated the decrease in mitochondrial membrane potential and Ca^2+^ disorder induced by doxorubicin in cardiomyocytes (Figures 6J–L). Taken together, GPX4 overexpression protected cardiomyocytes from doxorubicin-induced damage.

### 3.7 GPX4 served as a downstream target gene of β-catenin

**FIGURE 7:**
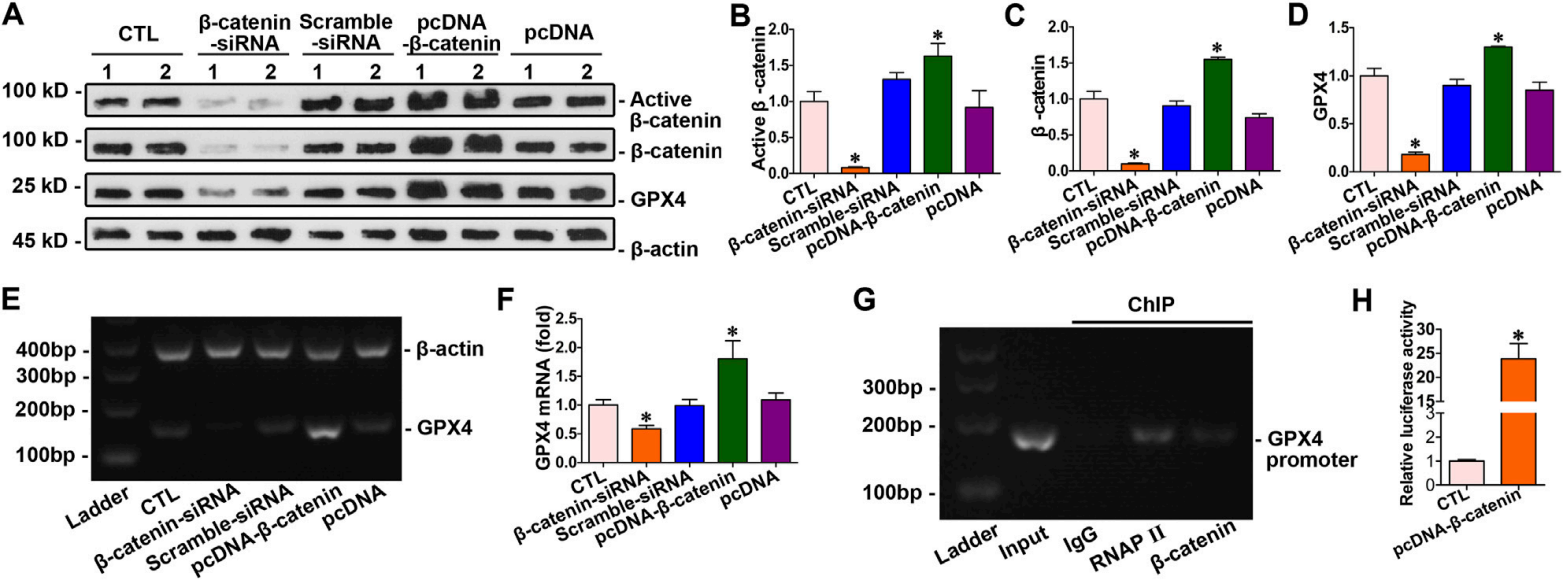
*GPX4* acted as a downstream target gene of β-catenin. **(A)** Western blot analysis revealed the expression levels of active β-catenin, total β-catenin, and GPX4 in cardiomyocytes with the overexpression or knockdown of β-catenin. **(B–D)** Quantitative analysis of the abundance of specific proteins in the indicated groups of Figure 7A is displayed. *P < 0.05 vs. the controls (n = 6). **(E)** PCR analysis of GPX4 mRNA levels following β-catenin gene overexpression and silencing. **(F)** Quantitative determination of the abundance of GPX4 mRNA levels in Figure 7E are presented. *P < 0.05 vs. the controls (n = 4). **(G)** ChIP assay verified that β-catenin bound to the specific site on the promoter of the *GPX4* gene. **(H)** Dual-luciferase reporter gene assay: both pGL6-TA and pRL-SV40-C were co-transfected into HEK293T cells with pcDNA3.1-β-catenin; both pGL6-TA and pRL-SV40-C were co-transfected into HEK293T cells in the control group. *P < 0.05 vs. the controls (n = 4).

The abovementioned findings indicated that β-catenin regulated doxorubicin-induced ferroptosis through GPX4 in cardiomyocytes. β-catenin expression was modulated via transfecting cardiomyocytes with pcDNA3.1–β-catenin or siRNA. The results showed that β-catenin overexpression led to an increase in both mRNA and protein levels of GPX4 in cardiomyocytes. Conversely, silencing β-catenin reduced the expression of GPX4 in myocytes (Figures 7A–F). Intervention experiments on β-catenin expression indicated that *GPX4* was a target gene of β-catenin. Moreover, ChIP assay combined with a dual-luciferase reporter gene assay confirmed the binding of β-catenin to the *GPX4* gene promoter. These findings suggested that *GPX4* functioned as a target gene regulated by β-catenin (Figures 7G,H).

## 4 Discussion

**FIGURE 8:**
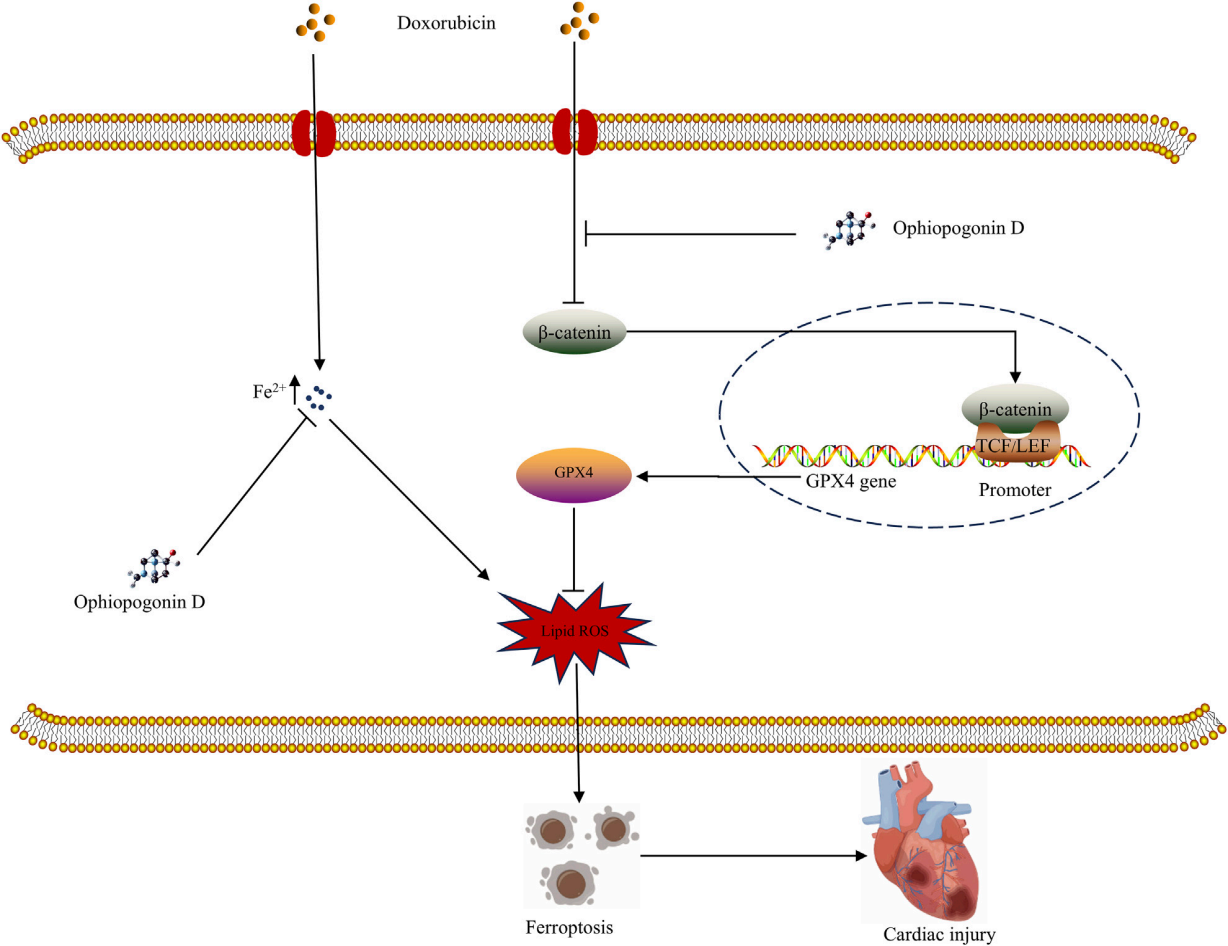
Graphical abstract of the mechanism underlying ophiopogonin D mitigating ferroptosis induced by doxorubicin.

Doxorubicin remains a cornerstone of cancer therapy. However, its cardiotoxicity not only causes significant patient suffering but also imposes a substantial economic burden on the healthcare system. The efficacy of protective drugs against doxorubicin-induced cardiotoxicity remains to be determined. A comprehensive investigation into the mechanisms underlying doxorubicin-induced cardiac damage, along with the development of corresponding cardioprotective drugs, has become an urgent and important topic to be addressed. Our study shows that doxorubicin induces ferroptosis in cardiomyocytes, while ophiopogonin D provides significant cardioprotection; the β-catenin/GPX4 signaling pathway plays an essential role in mediating this protective effect (Figure 8).

Ferroptosis represents a key mechanism through which doxorubicin causes cell damage (Yi et al., 2024). As a crucial inhibitor of ferroptosis, GPX4 inhibits ferroptosis by employing glutathione to reduce lipid ROS and alleviates mitochondrial oxidative stress (Fujii and Imai, 2024). Consistent with previous studies, doxorubicin results in a marked decrease of GPX4 levels in cardiomyocytes (TadokoroIkeda, 2020). Thus, doxorubicin may promote ferroptosis by suppressing the expression of GPX4 in cardiomyocytes. Moreover, GPX4 overexpression in cardiomyocytes markedly suppressed doxorubicin-induced ferroptosis, thereby confirming our speculation.

β-catenin is activated in tumor cells, whereas doxorubicin exerts its anti-tumor activity by suppressing the β-catenin signaling pathway (Sajeev et al., 2024). In normal cells, β-catenin signaling is maintained at a low baseline but is essential for physiological functions and cellular energy metabolism (Balatskyi et al., 2021). Surprisingly, doxorubicin significantly suppressed baseline β-catenin signaling in cardiomyocytes. Upon stimulation, such as by inflammation or oxidative stress, β-catenin signaling is crucial for facilitating cell survival and tissue repair (Jiang and Zhou, 2024; Vallée et al., 2021). It is important to note that β-catenin overexpression enhances GPX4 expression, thereby inhibiting doxorubicin-induced ferroptosis. Furthermore, ChIP and dual-luciferase reporter assays confirm that *GPX4* serves as a target gene of β-catenin and exerts anti-ferroptosis effects.

Shenmai injection, a Chinese herbal compound, has exhibited significant therapeutic efficacy in mitigating cardiotoxicity associated with anthracycline-based anti-cancer agents (Yang et al., 2024). Ophiopogonin D, an active constituent of the Shenmai injection, has been shown to enhance cardiac function in patients with heart failure (Wang et al., 2020).

We are greatly intrigued by whether ophiopogonin D can neutralize the cardiotoxicity of doxorubicin or not. Unexpectedly, ophiopogonin D demonstrates a significant capacity to attenuate cardiotoxicity associated with doxorubicin. Ophiopogonin D restores the baseline β-catenin signaling inhibited by doxorubicin, subsequently upregulating GPX4 expression and consequently inhibiting ferroptosis. Moreover, echocardiography revealed that doxorubicin markedly impaired the contractile function of the heart, whereas ophiopogonin D treatment conspicuously enhanced this function. With respect to the pharmacokinetics of ophiopogonin D, the results of animal experiments demonstrate that within the investigated concentration range, ophiopogonin D displays a good correlation coefficient. Following intravenous administration, its plasma concentration–time curve conforms to a two-compartment open model. Currently, the biological activities of ophiopogonin D encompass cardiovascular protection, immunomodulation, anti-inflammatory effects, anti-tumor properties, and bone protection (Chen et al., 2024). Ophiopogonin D constitutes one of the active components within Shenmai injection. The extensive clinical use of Shenmai injection offers a significant foundation for the clinical application feasibility of its principal active component, ophiopogonin D.

Divalent iron accumulation is a hallmark of ferroptotic cells. The present study finds that doxorubicin leads to a marked elevation of intracellular Fe^2+^, whereas ophiopogonin D and the overexpression of β-catenin or GPX4 were capable of reducing the level of Fe^2+^ within cardiomyocytes. In this regard, we have not conducted any further investigation into its specific mechanisms. This necessitates further exploration through additional experiments.

The research results demonstrate that ophiopogonin is capable of effectively counteracting cardiotoxicity induced by doxorubicin, with the mechanism of its action involving the suppression of ferroptosis. Furthermore, the specific mechanism by which ophiopogonin D inhibits ferroptosis has been preliminarily elucidated. Our findings offer new insights into the mechanisms of chemotherapy-induced cardiomyopathy and contribute to the development of cardioprotective therapies.

## 5 Conclusion

Ophiopogonin D restores β-catenin signaling and enhances the expression of the target gene *GPX4*, thereby inhibiting doxorubicin-induced ferroptosis in cardiomyocytes. The findings of this study suggest that ferroptosis is the key mechanism by which doxorubicin causes cardiotoxicity, and it also provides a promising strategy for the prevention of chemotherapy-related cardiomyopathy.

## Data Availability

The raw data supporting the conclusions of this article will be made available by the authors, without undue reservation.

## References

[B1] Balatskyi V. V. Vaskivskyi V. O. Myronova A. Avramets D. Abu Nahia K. Macewicz L. L. (2021). Cardiac-specific β-catenin deletion dysregulates energetic metabolism and mitochondrial function in perinatal cardiomyocytes. Mitochondrion 60, 59–69. 10.1016/j.mito.2021.07.005 34303005

[B2] Chen K. Q. Wang S. Z. Lei H. B. Liu X. (2024). Ophiopogonin D: review of pharmacological activity. Front. Pharmacol. 15, 1401627. 10.3389/fphar.2024.1401627 39101149 PMC11295246

[B3] Chen X. Cui H. Qin L. Liu R. Fang F. Wang Z. (2025). Soybean Lecithin-Gallic acid complex sensitizes lung cancer cells to radiation through ferroptosis regulated by Nrf2/SLC7A11/GPX4 pathway. Nutrients 17 (7), 1262. 10.3390/nu17071262 40219018 PMC11990552

[B4] Dadson K. Thavendiranathan P. Hauck L. Grothe D. Azam M. A. Stanley-Hasnain S. (2022). Statins protect against early stages of doxorubicin-induced cardiotoxicity through the regulation of Akt signaling and SERCA2. CJC Open 4 (12), 1043–1052. 10.1016/j.cjco.2022.08.006 36562012 PMC9764135

[B5] Dixon S. J. Lemberg K. M. Lamprecht M. R. Skouta R. Zaitsev E. M. Gleason C. E. (2012). Ferroptosis: an iron-dependent form of nonapoptotic cell death. Cell 149 (5), 1060–1072. 10.1016/j.cell.2012.03.042 22632970 PMC3367386

[B6] Fang X. Wang H. Han D. Xie E. Yang X. Wei J. (2019). Ferroptosis as a target for protection against cardiomyopathy. Proc. Natl. Acad. Sci. U. S. A. 116 (7), 2672–2680. 10.1073/pnas.1821022116 30692261 PMC6377499

[B7] Fujii J. Imai H. (2024). Oxidative metabolism as a cause of lipid peroxidation in the execution of ferroptosis. Int. J. Mol. Sci. 25 (14), 7544. 10.3390/ijms25147544 39062787 PMC11276677

[B8] Gessert S. Kühl M. (2010). The multiple phases and faces of wnt signaling during cardiac differentiation and development. Circ. Res. 107 (2), 186–199. 10.1161/circresaha.110.221531 20651295

[B9] Jiang Z. Zhou W. Tian X. Zou P. Zhang C. Li Y. (2024). A protective role of canonical wnt/β-catenin pathway in pathogenic bacteria-induced inflammatory responses. Mediat. Inflamm. 2024, 8869510. 10.1155/2024/8869510 PMC10914433 38445290

[B10] Keshavarzian E. Sadighpour T. Mortazavizadeh S. M. Soltani M. Motevalipoor A. F. Khamas S. S. (2023). Prophylactic agents for preventing cardiotoxicity induced following Anticancer agents: a systematic review and meta-analysis of clinical trials. Rev. Recent Clin. Trials 18 (2), 112–122. 10.2174/1574887118666230118102252 36803186

[B11] Lin S. Shi Q. Ge Z. Liu Y. Cao Y. Yang Y. (2021). Efficacy and safety of traditional Chinese medicine injections for heart failure with reduced ejection fraction: a Bayesian network meta-analysis of randomized controlled trials. Front. Pharmacol. 12, 659707. 10.3389/fphar.2021.659707 34916929 PMC8669995

[B12] Masuda T. Ishitani T. (2017). Context-dependent regulation of the β-catenin transcriptional complex supports diverse functions of Wnt/β-catenin signaling. J. Biochem. 161 (1), 9–17. 10.1093/jb/mvw072 28013224

[B13] Miller K. D. Nogueira L. Mariotto A. B. Rowland J. H. Yabroff K. R. Alfano C. M. (2019). Cancer treatment and survivorship statistics, 2019. CA Cancer J. Clin. 69 (5), 363–385. 10.3322/caac.21565 31184787

[B14] Renu K. V G. A. P B. T. Arunachalam S. (2018). Molecular mechanism of doxorubicin-induced cardiomyopathy - an update. Eur. J. Pharmacol. 818, 241–253. 10.1016/j.ejphar.2017.10.043 29074412

[B15] Reya T. Clevers H. (2005). Wnt signalling in stem cells and cancer. Nature 434 (7035), 843–850. 10.1038/nature03319 15829953

[B16] Sajeev A. Sailo B. Unnikrishnan J. Talukdar A. Alqahtani M. S. Abbas M. (2024). Unlocking the potential of Berberine: advancing cancer therapy through chemosensitization and combination treatments. Cancer Lett. 597, 217019. 10.1016/j.canlet.2024.217019 38849013

[B17] Singh K. A A. A. Ali Hamza A. M E. A.-G. Temurovich Islomov S. Fadhel Obaid R. (2023). Cardiac injury following chemo/radiation therapy: an updated review on mechanisms and therapeutic approaches. Curr. Radiopharm. 16 (3), 185–203. 10.2174/1874471016666230214101830 36786135

[B18] Tadokoro T. Ikeda M. Ide T. Deguchi H. Ikeda S. Okabe K. (2020). Mitochondria-dependent ferroptosis plays a pivotal role in doxorubicin cardiotoxicity. JCI Insight 5 (9), e132747. 10.1172/jci.insight.132747 32376803 PMC7253028

[B19] Valenta T. Hausmann G. Basler K. (2012). The many faces and functions of β-catenin. Embo J. 31 (12), 2714–2736. 10.1038/emboj.2012.150 22617422 PMC3380220

[B20] Vallée A. Vallée J. N. Lecarpentier Y. (2021). Potential role of cannabidiol in Parkinson's disease by targeting the WNT/β-catenin pathway, oxidative stress and inflammation. Aging (Albany NY) 13 (7), 10796–10813. 10.18632/aging.202951 33848261 PMC8064164

[B21] Wang J. You W. Wang N. Zhou W. Ge Y. Ma Z. (2020). Ophiopogonin D increases SERCA2a interaction with phospholamban by promoting CYP2J3 upregulation. Oxid. Med. Cell Longev. 2020, 8857906. 10.1155/2020/8857906 33488937 PMC7790559

[B22] Wu Y. P. Zhang S. Xin Y. F. Gu L. Q. Xu X. Z. Zhang C. D. (2021). Evidences for the mechanism of Shenmai injection antagonizing doxorubicin-induced cardiotoxicity. Phytomedicine 88, 153597. 10.1016/j.phymed.2021.153597 34111614

[B23] Yang L. Liu X. Yang W. Wang S. Li Z. Lei Y. (2024). Effect of shenmai injection on anthracycline-induced cardiotoxicity: a systematic review and meta-analysis. Complement. Ther. Med. 83, 103053. 10.1016/j.ctim.2024.103053 38801910

[B24] Yi X. Wang Q. Zhang M. Shu Q. Zhu J. (2024). Ferroptosis: a novel therapeutic target of natural products against doxorubicin-induced cardiotoxicity. Biomed. Pharmacother. 178, 117217. 10.1016/j.biopha.2024.117217 39079260

[B25] Zhao Y. Lei Y. Li Y. Zhang J. Tang H. (2021). Wnt/β‐catenin signalling mediates cardiac hypertrophy in type 4 cardiorenal syndrome. Nephrology 26 (6), 549–560. 10.1111/nep.13848

[B26] Zhou D. Yang Y. Han R. He J. Liu D. Xia W. (2024). Ferroptosis and its potential determinant role in myocardial susceptibility to ischemia/reperfusion injury in diabetes. Rev. Cardiovasc Med. 25 (10), 360. 10.31083/j.rcm2510360 39484139 PMC11522832

[B27] Zuo Y. Zhan L. Wen H. Xue J. Tan Y. Sun W. (2023). Stabilization of nuclear β-catenin by inhibiting KDM2A mediates cerebral ischemic tolerance. FASEB J. 37 (3), e22796. 10.1096/fj.202201657 36723950

